# Human Papilloma Virus Infection and Vaccination: Pre-Post Intervention Analysis on Knowledge, Attitudes and Willingness to Vaccinate Among Preadolescents Attending Secondary Schools of Palermo, Sicily

**DOI:** 10.3390/ijerph17155362

**Published:** 2020-07-25

**Authors:** Claudio Costantino, Emanuele Amodio, Francesco Vitale, Cecilia Trucchi, Carmelo Massimo Maida, Stefania Enza Bono, Francesca Caracci, Claudia Emilia Sannasardo, Francesco Scarpitta, Carlotta Vella, Gianmarco Ventura, Giancarlo Icardi, Alessandra Casuccio, Vincenzo Restivo

**Affiliations:** 1 Department of Health Promotion, Maternal and Infant Care, Internal Medicine and Excellence Specialties (PROMISE), University of Palermo, 90127 Palermo, Italy; claudio.costantino01@unipa.it (C.C.); emanuele.amodio@unipa.it (E.A.); carmelo.maida@unipa.it (C.M.M.); dr.stefaniabono@libero.it (S.E.B.); fracaracci@hotmail.it (F.C.); claudiaemilia.sannasardo@unipa.it (C.E.S.); francesco.scarpitta@unipa.it (F.S.); carlotta.vella@unipa.it (C.V.); gianmarco.ventura@unipa.it (G.V.); alessandra.casuccio@unipa.it (A.C.); vincenzo.restivo@unipa.it (V.R.); 2 A.Li.Sa. Liguria Health Authority, 16100 Genoa, Italy; cecilia.trucchi@regione.liguria.it; 3 IRCCS San Martino Hospital, 16100 Genoa, Italy; icardi@unige.it; 4 Department of Health Sciences, University of Genoa, 16100 Genoa, Italy

**Keywords:** HPV infection, HPV vaccine, knowledge, attitude, willingness, preadolescent students, vaccination, school-based intervention, sexually transmitted diseases

## Abstract

In recent years, vaccination coverage rates against Human Papilloma Virus (HPV) in Europe have shown a decreasing trend and remain below the required standard. The present study aims to assess knowledge and attitudes regarding HPV infection and vaccination among a representative sample of preadolescents of Palermo, Italy. A survey was carried out throughout two questionnaires, before and after carrying out an educational intervention scheduled during school hours. A total of 1702 students attending first-grade secondary schools of the province of Palermo were enrolled (response rate 68.9%). Students attending third classes (adj OR = 1.18; CI 95% 1.03–1.36), being of higher socioeconomic status (adj OR = 1.35; CI 95% 1.05–1.73), who had previously received information about sexually transmitted diseases (STDs) at home (adj OR = 1.62; CI 95% 1.27–2.07) or at school (adj OR = 2.15; CI 95% 1.70–2.71) and who had ever heard in the past about HPV (adj OR = 1.80; CI 95% 1.42–2.29) showed a significantly higher baseline level of knowledge regarding HPV. Willingness to receive HPV vaccination, in a 10-point Likert scale, significantly increased between the pre- (8.51; SD ± 1.79) and post- (9.01 SD ± 1.52) intervention questionnaires (*p* < 0.001). A total of 188 out of 272 (69.1%) preadolescents attending five out eighteen schools involved in the project, who had not previously received the HPV vaccine, were vaccinated. During past years, education campaigns on HPV were mainly dedicated to adult women, excluding teenagers and omitting young males. It should therefore be of primary importance that school-based education and vaccination programmes be standardized.

## 1. Introduction

The carcinogenic role of the Human Papilloma Virus (HPV) in the development of malignant tumors (cervical, ano-genital tract, and head and neck cancers) is widely recognized [1].

Several factors contribute to an increase in the risk of HPV infection, such as the number of previous and current sexual partners, the age of sexual debut, smoking attitudes, and congenital or acquired immunosuppression states [2].

Due to its infectious etiology, the main primary preventive strategy for HPV infection is represented by vaccination [3].

In several European countries, including Italy, HPV vaccination has been offered actively and free of charge to female subjects from 12 years old since 2008. Afterwards, the offer of a HPV vaccination was also extended to male preadolescents (from the age of 12 years old) since 2015 [4]. HPV vaccination catch-up campaigns were also encouraged, in co-payment, for all subjects of both sexes who had never been vaccinated (up to 45 years of age for females and up to 26 years for males) and for at-risk subjects (for example, the lesbian, gay, bisexual, and transgender community) [5,6].

Nevertheless, in recent years, vaccination coverage rates against HPV in numerous European countries have shown a decreasing trend among preadolescent females, and remain below the required standard [7]. In Italy, evaluating data from all 20 administrative regions, including Sicily, the vaccination adherence rate also remains considerably lower than the recommended 95% in the primary cohorts by the National Vaccination Plan 2017–2019 [8].

Moreover, there is a significant gap between females and males who received at least one dose and those who completed the vaccination schedule (two or three doses), imposing a careful evaluation of both organizational obstacles and reasons that determine the failure of vaccination schedule completion [9,10].

Several factors associated with poor knowledge and attitudes regarding HPV vaccination, such as the low education level of parents, not participating in school seminars on HPV, low risk-perception of the disease, and a limited knowledge of the effectiveness and safety of the HPV vaccination have been previously documented [11,12].

Objectives of the present study were to assess the baseline level of knowledge and attitudes regarding HPV infection and vaccination and the willingness to be vaccinated against HPV in a representative sample of preadolescents of both sexes attending secondary schools of Palermo; and after a formative intervention scheduled during school hours, to re-evaluate the knowledge on and willingness to undergo a HPV vaccination.

## 2. Materials and Methods

### 2.1. Study Design

A multicenter project coordinated by the Department of Health Sciences of the University of Genoa was conducted in four Italian Regions (Liguria, Veneto, Apulia, Sicily) with the main objective being to evaluate the offer of vaccination, coverage rates, and determinants associated with HPV vaccination uptake or refusal in different age classes and target groups [13,14,15].

Sicily is a southern Italian region with about five million inhabitants, and is fourth for demographic density in Italy [16]. Sicily is divided into nine Local Health Agencies (LHAs) that correspond to nine provinces (Agrigento, Caltanissetta, Catania, Enna, Messina, Palermo, Ragusa, Siracusa, Trapani). 

In particular, the province of Palermo and the corresponding LHA, accounting for 1,252,588 inhabitants residing in 82 municipalities, is the most populous in the region [16].

A pre–post interventional study evaluating knowledge and determinants associated with HPV vaccination in primary target cohorts (male and female preadolescents) was conducted in association with formative interventions in a sample of schools of the LHA of Palermo by healthcare professionals (medical researchers and postgraduate medical doctors) of the Department of Health Promotion, Maternal and Infant Care, Internal Medicine and Excellence Specialties of the University of Palermo, Italy, during two consecutive school years (2017/2018 and 2018/2019) in 18 first-grade secondary schools (accounting for 2469 students) located in Palermo.

The schools were sampled through cluster sampling based on urban location and were divided into three levels (from A: less deprived to C: more deprived), according to the deprivation index of the neighborhood or municipality in which they arose.

The deprivation index was calculated by using an algorithm including the average household income, the percentage of adults aged 25 with a high school diploma or university degree, and the percentage of employed people of the neighborhood or municipality of the first-grade secondary school [17].

In relation to the main outcome of the study (represented by the acceptability of the anti-HPV vaccination in the targets considered, that accounted for 50%) and to the population of preadolescents aged 11–14 years in the province of Palermo (51,888), 1492 preadolescents should be enrolled, so that the sample could be considered representative (95% confidence interval, margin error of 2.5% and adding a share of 5%, in consideration of the possible drop-outs and missed responses) [16].

An informative note on the objectives and purposes of the study was provided to all parents of the students attending the schools involved in the study, and a consent/dissent form was completed in order to agree to the participation to the survey and to the educational interventions on sexually transmitted diseases (STDs) and HPV vaccination.

During the 2018/2019 school year, with the logistical support of the Palermo LHA that provided material and human resources, the on-site HPV vaccination was contextually offered after the intervention in five schools in a dedicated and fully equipped caravan of the LHA, parked in the courtyards inside the schools.

### 2.2. Pre- and Post-Intervention Questionnaires

A survey was carried out throughout two (pre- and post-intervention) questionnaires, both administered in paper form, before and after carrying out an educational intervention on STDs, HPV infection, and preventive strategies.

The two questionnaires were borrowed from previous experience and tested for its comprehensibility and reproducibility on a sample of 102 preadolescents attending three first-grade secondary schools of Palermo.

The pre-intervention questionnaire included 15 items and was divided into two main sections: (1) a section included questions regarding socio-demographic characteristics and attitudes about STDs, particularly HPV; (2) a section aimed at investigating knowledge on HPV-related diseases and vaccination strategies, with five “sentinel” questions investigating HPV infection and vaccination (“Have you ever heard about HPV?”, “What is the principal way of transmission of HPV?”, “What are the target of HPV infections?”, “Can HPV infection cause genital warts?”, “What is the target for HPV vaccination?”).

A knowledge score, created for the study and tested in the analysis of the responses obtained by the first 102 students enrolled, that assigned one point for every correct response to the “sentinel” questions was calculated for every respondent. Specifically, students with three or more correct responses were assigned to the “adequate knowledge” group.

Moreover, a previously tested deprivation index was assigned to each participant in accordance with socio-demographic characteristics of students [17]. In particular, the educational qualification and work of the parents, family composition, and number of rooms available at home were included in the indicator [17].

From the sum of the obtained scores (from 0 to a maximum of 11), a range of values was defined, assigning to each student a deprived (from 0 to 5) or not deprived (from 6 to 11) status.

Furthermore, a post-interventional questionnaire included four items assessed, through a Likert scale (1 points: Strongly Agree, 2 points: Agree, 3 points: Neutral, 4 points: Disagree, 5 points: Strongly Disagree), knowledge about HPV infection or HPV vaccination before the seminars.

Finally, a 10-point Likert scale question on how much the participant was willing to receive the HPV vaccine was included both in the pre- and post-intervention questionnaires, with the aim to evaluate the effectiveness of the intervention.

### 2.3. Educational Intervention and On-Site HPV Vaccination Offer

The educational interventions for preadolescents included a frontal lesson of about 20 min, conducted by medical doctors and researchers of the Department of Health Promotion, Maternal and Infant Care, Internal Medicine and Excellence Specialties of the University of Palermo.

In supporting the intervention, a set of slides relating to STD prevention with a particular focus on HPV infection, related diseases, and vaccination was presented in plenary sessions to all students for which the consent form signed by parents was obtained.

The personal data and the results of the pre- and post-interventional questionnaires were collected in accordance with the rules of good clinical practice, and each questionnaire was anonymous.

In order to verify that each student completed both questionnaires and that the pre- and post-educational intervention willingness to HPV vaccination might be compared, all participants were provided two unique anonymous codes that were used at the beginning of both questionnaires.

The study protocol was authorized and approved by the Regional Ethics Committee of the Liguria Region (Protocol number 162 REG 2017 or 11 April 2017) and by the Ethical Committee Palermo 1 (Protocol number 6/2017 of 5 June 2017).

### 2.4. Statistical Analysis

Data collected through written questionnaires were entered into a database created with EpiInfo 3.5.4 (Centers for Disease Control and Prevention, Atlanta, GA, USA). All the data were analyzed using the statistical software package Stata/MP 12.1 (StataCorp LP, College Station, TX, USA).

Absolute and relative frequencies were calculated for the categorical (qualitative) variables. The differences in the categorical variables for hesitancy and refusal and between before and after the intervention were analyzed using chi-squared tests (Mantel–Haenszel) and the McNemar test, respectively. 

Correct responses to at least three of the five “sentinel” questions investigating HPV infection and vaccination knowledge was considered as a dependent variable (good knowledge) in the uni/multivariate analysis conducted with principal variables examined in the pre-intervention questionnaire.

Variables found to have a statistical association with a *p*-value ≤ 0.20 at the univariate analysis, to guarantee a more conservative approach, were included in the two multivariate backward stepwise logistic regression models carried out. The crude odds ratio (crude OR) and the adjusted OR (adj-OR) with 95% confidence intervals (CIs) were calculated in the logistic regression models. A *p*-value ≤ 0.05 was considered significant throughout the study.

## 3. Results

A total of 1702 students attending first-grade secondary schools of the province of Palermo were enrolled (response rate 68.9%), of which 52% were female. About half of the preadolescents (47.9%) attended third classes, and 50.4% belonged to schools of an intermediate level (B) of neighborhood socioeconomic index, followed by students belonging to schools of high (A = 25.7%) and low levels (C = 24%). A large majority of respondents (98.1%) were Italian, and 64.2% attended schools in the Municipality of Palermo. Finally, regarding the deprivation index, 67.1% of enrolled students reported a deprived socio-economic status (Table 1).

A large majority of students had already attended educative lessons about STDs at school (66.5%), while only 32.4% of respondents previously had information about STDs at home, and 21.7% with at least one physician. Moreover, 71.3% of the sample had previously heard about the HPV infection or vaccine and 88% knew the correct way of HPV transmission (sexual intercourse) (Table 2). Almost half of the students enrolled (50.2%) was aware of the possibility that HPV could infect both sexes, and 83.4% of the sample knew that HPV vaccination in Sicily was offered to both male and female preadolescents. Furthermore, only 39% and 17.7% of the students enrolled were aware that HPV infection was common and could cause genital warts, respectively (Table 2).

In Table 3, crude ORs and adj ORs of variables associated with adequate knowledge regarding HPV infection and vaccination are reported. In particular, students attending third classes (adj OR = 1.18; CI 95% 1.03–1.36), who were of not deprived socioeconomic status (adj OR = 1.35; CI 95% 1.05–1.73), who had previously received information about STDs at home (adj OR = 1.62; CI 95% 1.27–2.07) or at school (adj OR = 2.15; CI 95% 1.70–2.71), and who had ever heard in the past about HPV (adj OR = 1.80; CI 95% 1.42–2.29) showed a significantly higher level of knowledge regarding HPV infection and vaccination.

In Table 4, the answers to four questions investigating students’ knowledge on HPV infection and vaccination of the post-intervention questionnaire are reported. A total of 1605 students responded to the post-intervention questionnaire (*n* = 97 students did not participate in the pre–post interventional studies). Specifically, about 85% of students participating to the formative intervention and responding to the post-intervention questionnaire provided a correct response to the questions.

Willingness to receive HPV vaccination, in a 10-point Likert scale, among students enrolled in the study significantly increased between the pre- (8.51; SD ±1.79) and post- (9.01 SD ±1.52) intervention questionnaires (+0.50; *p* < 0.001; data not shown in figure).

Table 5 shows the crude ORs and adj ORs of variables associated with an increase in willingness to receive HPV vaccination after the intervention was conducted at schools. Female students (adj OR = 1.66; CI 95% 1.20–2.31) and students attending schools in the municipality of Palermo (adj OR = 1.55; CI 95% 1.09–2.22) showed a significant increase in willingness.

Finally, in 5 out 18 schools that met all the authorization and safety criteria, HPV vaccination was offered to all students not already vaccinated. A total of 188 out of 272 (69.1%) preadolescents that participated in the formative interventions and that had not previously received at least one dose of HPV vaccination were vaccinated against HPV after the participation in the formative interventions, in collaboration with medical officers of the LHA of Palermo and after the collection of consent forms signed by parents (data not shown).

## 4. Discussion

In Italy, only a few studies evaluating the main reasons associated with low HPV vaccination knowledge and attitudes were conducted during recent years [18,19].

Among preadolescents and their parents, one of the main reasons was represented by the role of the family paediatricians or the general practitioners in recommending the vaccination to parents [11,18].

Results of the present study, on the other hand, demonstrate that better attitudes and knowledge regarding HPV infection and vaccination are attributable to the formative role of families and schools. Students that had previously received information about STDs at home or at school showed statistically significantly higher knowledge scores on HPV than others.

Formative intervention conducted at school was strongly associated with HPV vaccination knowledge and uptake and, for instance, female young adults who did not participate in school seminars on HPV demonstrated lower vaccination uptake rates in previous studies [9,20,21].

A systematic review also confirmed that the best strategies to increase attitudes regarding HPV vaccination were both parental education (through brochures and information sheets or invitation letters) and adolescents’ education (through, for example, frontal lessons or educational messages conveyed by the most used social media) [22].

This could explain data obtained among students of the Palermo province, in which better knowledge on HPV infection and vaccination were observed among students with a low deprivation index. In general, minor attention given to informative and educative messages and a better response to invitation letters sent by LHAs were shown by most deprived subjects in Sicily [23,24].

In order to improve vaccination awareness and adherence among deprived social classes, school seminars and vaccination offer could probably be considered effective interventions aimed at improving the adherence to HPV vaccination [25].

According to results obtained in the present study, satisfying data were reported in the sentinel questions regarding HPV infection and vaccination knowledge of the post-intervention questionnaire. The willingness to receive HPV vaccination also showed a statistically significant 6% increase.

Usually, information and recommendations provided by healthcare professionals can be considered one of the strongest predictive factors by increasing the chances of adherence to HPV vaccination [26]. However, in preadolescents and school-aged children, the support of parents and of educative seminars conducted at school could have a better impact in improving knowledge and attitudes regarding preventive issues, as showed in the study sample, supporting the role of school intervention on STDs and HPV [27,28].

During past years, education campaigns on HPV were mainly dedicated to adult women, excluding teenagers, and systematically omitted young males [12].

In the study sample, an increased willingness to receive HPV vaccination was observed among female and male students attending schools of the Palermo municipality. Generally, these findings could be attributed to major attention of these students given to personal risk perception of contracting the infection (female) or better school education due to the lower number of students in classrooms, which is more frequent in the schools of the Palermo municipality [29,30].

Finally, parental awareness represented one of the main factors influencing the acceptance of the HPV vaccine among preadolescents, and was associated with the quantity and quality of information provided on HPV, also at school [31].

It should therefore be of primary importance that medical and sanitary teams support and carry out school-based education and vaccination programmes about teenagers’ sexuality, STDs, and preventive strategies, such as HPV vaccination.

In particular, students attending schools in more deprived areas or students with poor socio-economic conditions are least informed about STDs or the opportunity to protect themselves with vaccination. For these reasons, the school environment should represent an optimal place for sharing correct information and to improve knowledge and attitudes on STDs prevention, regardless of personal or school deprivation index [32].

As a matter of fact, during the study duration, 188 preadolescent students of five schools (69.1% of the susceptible sample) not already immunized against HPV were vaccinated, demonstrating the high efficacy of school-based formative intervention and a vaccination offer.

Some limitations could have affected the present study. First, a selection bias should be considered, since greater participation of students whose parents were more prone to vaccine compliance could not be excluded. Secondly, the study was based on self-reported information of a representative sample of preadolescents—although self-reporting can be a risk for inaccuracy, it was recognized as a cost-effective and feasible method for gathering data from large population samples, also among secondary school students [33]. Finally, another important limitation of this study was related to the study design (before/after design without randomization and group control) that was purely descriptive and did not allow for important fact-based evidence.

## 5. Conclusions

HPV vaccination represents a clear example of under-use of a practice with a very high scientific value [7]. In Italy, vaccination coverage rates among preadolescents remain considerably low [8]. Of note, the future role of parents and of school educational intervention could represent a solution to improve vaccination attitudes and knowledge of preadolescents, that represents the primary target of HPV vaccination [34].

The large-scale organization of school-based educational interventions on STDs, HPV-related diseases and preventive strategies should probably be standardized and extended in order to improve awareness and willingness of students on the importance of HPV vaccination.

## Figures and Tables

**Table 1:** Sociodemographic and general characteristics of students enrolled in the first-grade secondary schools of Palermo, in the pre-intervention questionnaire (*n* = 1702).

Variables	*n* (%)
**Gender**	
Male	802 (48)
Female	869 (52)
**School year**	
first class	390 (22.9)
second class	497 (29.2)
third class	815 (47.9)
**School neighborhood deprivation index**	
A (high level)	437 (25.7)
B (intermediate level)	858 (50.4)
C (low level)	407 (23.9)
**Residency**	
Palermo municipality	1092 (64.2)
Province of Palermo	610 (35.8)
**Nationality**	
Italian	1622 (98.1)
Other	31 (1.9)
**Deprivation index**	
Deprived	1138 (67.1)
Not Deprived	559 (32.9)

**Table 2:** Attitudes and knowledge of students enrolled in the study, regarding sexually transmitted diseases (STDs) and Human Papilloma Virus (HPV) vaccination, in the pre-intervention questionnaire (*n* = 1702).

Variables	*n* (%)
**Talking about STDs at home**	
Yes	551 (32.4)
No	1151 (67.6)
**Talking about STDs at school**	
Yes	1131 (66.5)
No	571 (33.5)
**Talking about STDs with physician**	
Yes	370 (21.7)
No	1332 (78.3)
**Have you ever heard about HPV infection or vaccine**	
Yes	1214 (71.3)
No	488 (28.7)
**Principal way of transmission of HPV infection**	
Sexual intercourse	1497 (88)
Airborne	147 (8.6)
Oro-fecal	58 (3.4)
**HPV infection is common**	
Yes	703 (39)
No	1098 (61)
**HPV infection can cause cancer among both sexes**	
Yes	905 (50.2)
No	896 (49.8)
**HPV infection can cause genital warts**	
Yes	319 (17.7)
No	1482 (82.3)
**HPV vaccination is actively and free of charge offered to**	
Female preadolescents	299 (16.6)
Both Sexes	1502 (83.4)

**Table 3:** Crude OR and Adjusted OR of variables associated with adequate knowledge (score ≥ 3 in the items investigating knowledge about HPV infection and HPV vaccination) among students enrolled before the intervention conducted.

Variables		Crude OR	CI 95%	*p*-Value	Adj OR	CI 95%	*p*-Value
**Gender**	Male	ref		0.14	ref		0.36
	Female	1.16	0.95–1.42		1.11	0.89–1.37	
**School year**	First	ref		<0.001	ref		0.01
	Second	1.95	1.49–2.54		1.09	0.98–1.95	
	Third	2.11	1.68–2.66		1.18	1.03–1.36	
**School Deprivation Index**	A	ref		0.04	ref		0.75
	B	1.01	0.76–1.18		1.03	0.87–1.21	
	C	0.66	0.53–0.91		0.89	0.72–1.06	
**Residency**	Province	ref		0.48			
	Municipality	0.95	0.89–1.27				
**Nationality**	Italian	ref		0.92			
	Other	1.04	0.49–2.18				
**Deprivation Index**	Deprived	ref		<0.001	ref		0.02
	Not Deprived	1.60	1.29–2.00		1.35	1.05–1.73	
**Talking about STDs at home**	No	ref		<0.001	ref		<0.001
	Yes	2.13	1.70−2.67		1.62	1.27–2.07	
**Talking about STDs at school**	No	ref		<0.001	ref		<0.001
	Yes	2.65	2.15–3.27		2.15	1.70–2.71	
**Talking about STDs with physician**	No	ref		0.38			
	Yes	1.11	0.87–1.42				
**Have you ever heard about HPV infection or vaccine**	No	ref		<0.001	ref		<0.001
	Yes	2.34	1.88–2.90		1.80	1.42–2.29	

**Table 4:** Knowledge of students enrolled in the study regarding HPV infection and vaccination in the post-intervention questionnaire (*n* = 1605).

Questions	Correct Answer	Uncorrect Answer
	***n* (%)**	
HPV infection can cause severe diseases (such as cancers)	1504 (93.7)	101 (6.3)
HPV vaccination can prevent cervical cancer, ano-genital cancers and genital warts	1445 (90.2)	157 (9.8)
HPV vaccination is effective and safe	1379 (85.9)	226 (14.1)
Preadolescent boys and girls should be vaccinated against HPV	1450 (90.5)	153 (9.5)

**Table 5:** Crude ORs and Adjusted ORs of variables associated with an increase in willingness to receive HPV vaccination after the intervention conducted at schools.

Variables		Crude OR	CI 95%	*p*-Value	Adj OR	CI 95%	*p*-Value
**Gender**	Male	ref		<0.001	ref		<0.01
	Female	1.69	1.23–2.55		1.66	1.20–2.31	
**Nationality**	Italian	ref		0.63			
	Other	1.13	0.86–1.69				
**School year**	First	ref		0.81			
	Second	0.87	0.64–1.26				
	Third	1.17	0.89–1.67				
**School Deprivation Index**	A	ref		<0.05	ref		0.10
	B	1.16	0.76–1.18		1.02	0.89–1.12	
	C	1.34	1.09–1.72		1.20	0.96–1.49	
**Residency**	Province	ref		<0.05	ref		<0.05
	Municipality	1.54	1.09–2.19		1.55	1.09–2.22	
**Deprivation** **Index**	Deprived	ref		0.36			
	Not Deprived	0.93	0.67–1.29				
**Have you ever heard about HPV infection or vaccine**	No	ref		<0.01	ref		0.07
	Yes	1.89	1.23–2.19		1.36	0.97–1.95	
**Knowledge score at preintervention questionnaire**	Not adequate	ref		0.09	ref		0.14
	Adequate	1.45	0.96–1.93		1.28	0.92–1.79	
